# Preparation and Properties of Calcium Peroxide/Poly(ethylene glycol)@Silica Nanoparticles with Controlled Oxygen-Generating Behaviors

**DOI:** 10.3390/ma18112568

**Published:** 2025-05-30

**Authors:** Xiaoling Xie, Xin Sun, Wanming Lin, Xiaofeng Yang, Ruicong Wang

**Affiliations:** 1 College of New Energy and Materials Engineering, Shanxi University of Electronic Science and Technology, Linfen 041000, China; xiexl2003@126.com (X.X.); yangxiaofeng@sxdzkj.edu.cn (X.Y.); wangruicong@sxdzkj.edu.cn (R.W.); 2 Zhejiang Institute of Tianjin University, Ningbo 315201, China; sunxin_tju@163.com

**Keywords:** poly (ethylene glycol), calcium peroxide, oxygen generating, biocompatibility, silica shell

## Abstract

The hypoxic microenvironment is the main challenge for the repair of damaged tissue, and oxygen supply is an effective means of alleviating hypoxia. In this study, a series of core–shell-structured calcium peroxide/poly(ethylene glycol)@silica (CPO@SiO_2_) nanoparticles are prepared to generate oxygen steadily. The size of the CPO@SiO_2_ nanoparticles ranges from 205 to 302 nm, with a narrow polydispersity index (PDI). In this system, the nano CPO core acts as the oxygen source to improve hypoxia, while the SiO_2_ shell layer serves as the physical barrier to control the oxygen-generating rate and improve biocompatibility. The results suggest that the thickness of the SiO_2_ shell layer can be modulated by adjusting the amount of tetraethyl orthosilicate (TEOS). The prepared CPO@SiO_2_ nanoparticles show a controlled oxygen-generating rate. Moreover, compared with CPO, the CPO@SiO_2_ nanoparticles have good biocompatibility. To assess the modulating effects for the hypoxic microenvironment, L929 cells are co-cultured with CPO@ SiO_2_ nanoparticles under hypoxia. The results suggest that the CPO@ SiO_2_ nanoparticles can support the cell survival under hypoxia. Moreover, they can effectively decrease oxidative stress damage and reduce the levels of expression of hypoxia-induced superoxide dismutase (SOD) and malondialdehyde (MDA). Therefore, the prepared CPO@ SiO_2_ nanoparticles with controlled oxygen-generating properties could be a promising candidate for repairing damaged tissue.

## 1. Introduction

Oxygen is essential to cell survival and tissue function [1]. In pathological conditions, hypoxia frequently occurs in damaged tissues, including myocardial infarction [2,3], nerve injury [4,5], and skin wounds [6,7]. The hypoxic state triggers oxidative stress, induces cell dysfunction or even necrosis, and substantially impairs tissue repair. Moreover, inadequate oxygen supply poses a major challenge in tissue engineering, where cells in the central regions of engineered constructs typically exhibit oxidative stress damage, reduced survival, and suppressed proliferation [8,9]. Therefore, developing effective oxygen delivery strategies is essential for alleviating hypoxia and promoting tissue regeneration.

Various oxygen delivery systems have been developed to address hypoxia, including direct oxygen supply, perfluorocarbon/hemoglobin-based oxygen delivery, and oxygen-generating biomaterials [10]. However, conventional direct oxygen delivery is inherently unsustainable and may cause excessive oxygen accumulation in healthy tissues, which could lead to reactive oxygen species (ROS) production and subsequent cell death [11]. Therefore, precise control of oxygen supply rates is critical to enhancing cell survival and functional performance under hypoxia. Perfluorcarbon (PFC)-based composites are usually employed as oxygen carriers by increasing local dissolved oxygen levels [12]. For instance, Niu et al. developed a methacrylate-poly (ethylene glycol) (PEG)-perfluorooctane hydrogel with a high oxygen-retention capacity to support the survival and proliferation of bone-marrow mesenchymal stem cells under hypoxia [13]. Nevertheless, this approach is constrained by three major limitations: limited oxygen-carrying capacity, uncontrolled release behavior, and potential toxicity [10]. For long-term applications, oxygen-generating materials represent a more suitable alternative. Hydrogen peroxide (H_2_O_2_) can directly generate an oxygen source through decomposition into water and oxygen [11]. However, H_2_O_2_-based systems often suffer from rapid oxygen release kinetics due to their high water solubility and decomposition rate [14]. In addition, direct cellular exposure to H_2_O_2_ may trigger oxidative stress damage.

Calcium peroxide (CaO_2_, CPO) can generate O_2_ by forming H_2_O_2_ following its hydrolysis. It is an effective solid-oxygen-generating material that can stably generate O_2_ via reacting with water in vivo and in vitro, supporting cell proliferation and differentiation [15], and providing oxygen for tissue-engineered implants before capillary network formation [16]. Some polymers (e.g., PEG [17,18], polydimethylsiloxane [19,20], alginate [12,21,22,23,24], gelatin [25,26,27], etc.) are incorporated with CPO through composite or coating strategies to modulate the oxygen production rate and biocompatibility. For example, in bone tissue engineering [28,29,30,31], Hsieh et al. designed CPO/MnO_2_-encapsulated poly (lactic-co-glycol acid) (PLGA) microspheres as an oxygen-generating system for local oxygen delivery to promote bone regeneration [32]. However, most reported CPO particles are micron-sized [12,21,26,33,34,35,36] and tend to agglomerate and distribute homogeneously in scaffold networks. Although the reaction rate of CPO with water is lower than H_2_O_2_, it remains too rapid compared to the rate of tissue repair and engineered tissue formation [37]. Adjusting the reaction rate of CPO with water is an effective method to control the rate of oxygen generation and prolong the process of oxygen release. Adding a coating shell layer onto CPO to prepare core–shell nanoparticles is an effective way to modulate the reaction of CPO with water [38]. Recent comprehensive reviews have systematically documented the evolving fabrication strategies for calcium-peroxide-based biomedical platforms, particularly highlighting the structural advantages of core–shell architectures in controlled oxygen release [39]. Silica (SiO_2_) is an ideal shell layer that enhances CPO dispersion in aqueous systems and controls the oxygen generation via physical barrier effects [40,41]. Wu et al. designed CaO_2_@SiO_2_-PAA nanoparticles by encapsulating CaO_2_ in hollow mesoporous SiO_2_ and coating with polyacrylic acid (PAA). The system could efficiently deliver CaO_2_ to the tumor site and release ROS [42]. However, few studies have focused on controlling oxygen generation in CaO_2_-based core–shell architectures.

This study aims to develop nanoparticles with tunable oxygen-generating properties to reduce oxidative stress damage in hypoxic cells through sustained oxygen supply. To achieve this goal, we engineered core–shell structured CPO@SiO_2_ nanoparticles. The SiO_2_ shell was coated on the nano CPO cores to construct these CPO@SiO_2_ nanoparticles, which exhibited uniform dispersion. Moreover, the nano CPO core functions as an oxygen source to alleviate hypoxia, while the SiO_2_ shell acts as a physical barrier that (1) regulates the oxygen generation rate by controlling the reaction between the CPO core and water; (2) reduces cytotoxicity from excessive H_2_O_2_ production; and (3) enhances nanoparticle biocompatibility. Therefore, the prepared CPO@SiO_2_ could improve cell survival and proliferation under hypoxia and reduce oxidative stress damage. The thickness of the SiO_2_ shell layer can be modulated by adjusting the amount of tetraethyl orthosilicate (TEOS). The chemical structure, morphology, size, and oxygen-generating behaviors of the prepared CPO@SiO_2_ nanoparticles were systematically characterized. Subsequently, their biocompatibility was evaluated using L929 cells. Finally, the effects of the CPO@SiO_2_ nanoparticles on the cell survival of and oxidative stress suppression were assessed under hypoxia.

## 2. Materials and Methods

### 2.1. Materials

Catalase (3500 units/mg) was obtained from Meryer (Shanghai, China). Poly (ethylene glycol) (PEG, Mw = 200), tetraethyl orthosilicate (TEOS), calcium chloride (CaCl_2_), ammonia, and hydrogen peroxide solution (30% *w*/*w*) were purchased from Aladdin (Shanghai, China). Potassium permanganate (KMnO_4_), sodium oxalate, and sulfuric acid were obtained from Jiangtian Chemical Technology Co., Ltd., Tianjin, China. All other chemicals were analytic grade and used without further purification.

### 2.2. Preparation of CPO@SiO_2_ Nanoparticles

Calcium peroxide (CPO) nanoparticles were prepared in the presence of PEG according to Equation (1):

CaCl_2_ + H_2_O_2_ + 2NH_3_·H_2_O → CaO_2_ + 2NH_4_Cl + 2H_2_O (1)

Briefly, 3 g of calcium chloride (CaCl_2_) was dissolved in 30 mL of distilled water, followed by the addition of 15 mL of ammonia (30 wt%) under stirring. Subsequently, 120 mL of PEG was added as a dispersant. Then, 15 mL of hydrogen peroxide (30% *w*/*w*) was added dropwise at a controlled rate of 0.25 mL/min with continuous stirring for 1 h. This reaction was maintained for 2 h, after which the pH was adjusted to 11.5 using 0.1 M NaOH solution. After sedimentation, the supernatant was carefully removed, and the precipitate was washed three times with 0.01 M NaOH solution. Finally, CPO nanoparticles were obtained through freeze-drying under optimized conditions, with the cold trap temperature maintained at −60 °C and a system vacuum below 10 Pa.

To fabricate the SiO_2_ layer on the nano CPO surface, 50 mg of CPO was dispersed in 12 mL of ammonia solution (28% *w*/*w*) and 100 mL of ethanol. After 30 min of ultrasonication, tetraethyl orthosilicate (TEOS) was added dropwise at the rate of one drop per 6 min under stirring. The reaction was carried out at room temperature for 6 h. After aging overnight, the solid precipitate was washed three times with 0.4% acetic acid solution. The final core–shell-structured CPO@SiO_2_ nanoparticles were collected via centrifugation and freeze-drying. The thickness of the SiO_2_ layer was modulated by varying the TEOS volume (2, 1, 0.5, 0.3, 0.2, and 0.1 mL), and the corresponding samples were designated as CPO@SiO_2_-2.0T, CPO@SiO_2_-1.0T, CPO@SiO_2_-0.5T, CPO@SiO_2_-0.3T, CPO@SiO_2_-0.2T, and CPO@SiO_2_-0.1T, respectively.

### 2.3. Characterizations

The chemical structures of the prepared CPO and CPO@SiO_2_-1.0T samples were investigated using Fourier transform infrared spectroscopy (FTIR) (Nicolet 6700, Thermo Fisher, Waltham, MA, USA) in the range of 4000–500 cm^−1^. The X-ray diffraction (XRD) patterns of CPO and CPO@SiO_2_-1.0T were collected over a 2θ range of 10° to 80° with a scan rate of 8.0°/min at 36 kV and 200 mA.

The hydrodynamic sizes of the CPO@SiO_2_ were measured using a Zetasizer Nano ZS, (Malvern Instruments, Malvern, UK) at 25 °C with a detection angle of 173°. Briefly, the prepared CPO and CPO@SiO_2_ nanoparticles were dispersed in ethanol at a concentration of 1 mg/mL using ultrasonication (100 W, 15 min). The particle size distribution was measured using a Malvern Nano Zeta laser particle size analyzer (Malvern Instruments, Malvern, UK).

The morphology of CPO@SiO_2_ was evaluated using scanning electron microscopy (SEM) at an accelerating voltage of 5.0 kV and transmission electron microscope (TEM) at an accelerating voltage of 200 kV, with the elemental mapping of Si, O, and Ca performed using TEM-EDS.

### 2.4. Determination of CPO Contents

The CaO_2_ contents in the CPO@SiO_2_ nanoparticles were determined via potassium permanganate titration. The procedure was as follows: first, a 0.02 M potassium permanganate (KMnO_4_) aqueous solution was prepared. The KMnO_4_ solution was standardized by titration against sodium oxalate (0.15–0.2 g, pre-dried at 120 °C for ≥1 h) dissolved in 1 M sulfuric acid, with the reaction carried out in a water bath maintained at 80–90 °C. For the sample analysis, the same titration procedure was performed using CPO@SiO_2_-2.0T, CPO@SiO_2_-1.0T, CPO@SiO_2_-0.5T, or CPO@SiO_2_-0.3T nanoparticles instead of sodium oxalate. The CaO_2_ content in each sample was calculated based on the titration results. The relevant reaction equation is provided below:

2MnO_4_^−^ + 5C_2_O_4_^2−^ + 16H^+^ = 2Mn^2+^ + 5CO_2_↑ + 8H_2_O (2)

CaO_2_ (s) + 2H_2_O → Ca(OH)_2_ (s) + H_2_O_2_ (3)

2MnO_4_^−^ + 5H_2_O_2_ + 6H^+^ = 2Mn^2+^ + 5O_2_↑ + 8H_2_O (4)

### 2.5. The Oxygen Generation Assay

**Figure 1:**
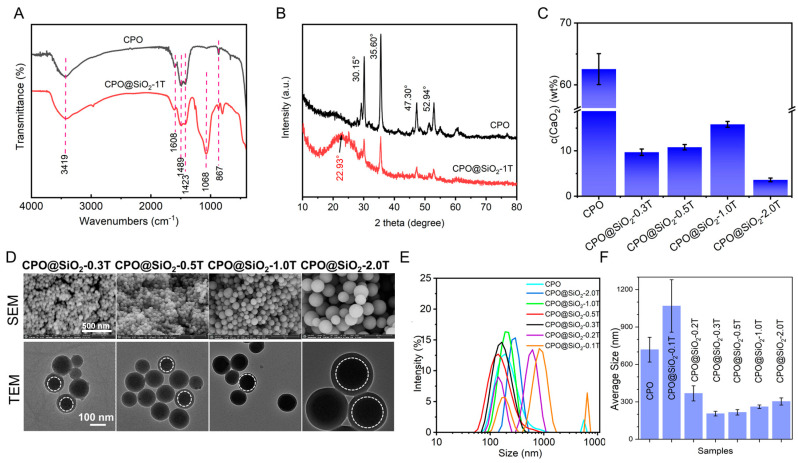
Characteristics and morphology of CPO@SiO_2_ nanoparticles. (**A**) FT-IR spectra and (**B**) XRD patterns of CPO and CPO@ SiO_2_-1.0T. (**C**) Calcium peroxide content in CPO and CPO@SiO_2_. (**D**) SEM and TEM images of CPO@SiO_2_-0.3T, CPO@SiO_2_-0.5T, CPO@SiO_2_-1.0T, and CPO@SiO_2_-2.0T. (**E**) Size curves and (**F**) average sizes of different CPO@ SiO_2_ nanoparticles measured using DLS.

First, 50 mL of catalase solution (100 U/mL) was added to a high-pressure airtight container, and dissolved oxygen was purged using nitrogen. Then, predetermined amounts of CPO or CPO@SiO_2_ with varying shell thicknesses and concentrations were added. Oxygen content was detected using a PreSens Headspace Residual Oxygen Analyzer (Microx 4 Trace, PreSens, Regensburg, Germany), with dissolved oxygen (DO) (mg/L) measurements taken at designated time points. In order to explore the effect of different shell thicknesses while maintaining consistent total oxygen release across samples, we normalized the nanoparticle concentrations based on their CaO_2_ content (Figure 1C), This normalization ensured that all experimental groups (except the control and CPO@SiO_2_-1.0T groups) were adjusted to concentrations that would generate oxygen equivalent to 0.1 mg/mL of CPO@SiO_2_-1.0T for all experimental groups. Furthermore, equivalent concentrations (0.02, 0.06, and 0.1 mg/mL) of CPO@SiO_2_-1.0T were tested to evaluate the CPO@SiO_2_ oxygen-generation profiles.

### 2.6. In Vitro Cytotoxicity Assay

L929 cells, widely used as a model for evaluating biomaterial cytotoxicity and cell–biomaterial interactions, were employed to assess the CPO@SiO_2_’s biocompatibility. The experimental procedure was conducted as follows: first, L929 cells were seeded into 96-well plates at a density of 1 × 10^4^ cells/well and cultured for 6 h in RPMI-1640 medium containing 10 wt% fetal bovine serum (FBS) and a 1% mixture of penicillium and streptomycin. Then, the culture medium was replaced with 200 μL of fresh culture medium containing different concentrations of CPO@SiO_2_, followed by 24 h incubation. After incubation, 20 μL of Cell Counting Kit-8 (CCK-8) solution was added to each well and incubated for another 2 h at 37 °C. The absorbance of the supernatant was measured at 450 nm using a microplate reader (PerkinElmer, Phoenix, AZ, USA), with the cell culture medium as the blank and untreated normal cells as the control. The cell viability was calculated using Equation (5):

(5) $$\mathrm{Cell}\;\mathrm{viability}\;(\%)=\frac{\left(OD_{sample}-OD_{blank}\right)}{\left(OD_{control}-OD_{blank}\right)}\times 100\%$$

The cellular activity and morphology of L929 cells treated with CPO@SiO_2_ were also investigated using acridine orange/propidium iodide (AO/PI) staining. Fluorescent images were acquired using an IX-51 fluorescence microscope (Olympus, Tokyo, Japan).

### 2.7. Detection of Reactive Oxygen Species in Cells

L929 cells were seeded in 96-well plates at a density of 5 × 10^3^ cells/well and cultured at 37 °C under normoxic conditions (21% O_2_ and 5% CO_2_). After 6 h, the culture medium was replaced with 200 μL of fresh medium containing CPO@SiO_2_, and cells were transferred to a hypoxia incubator (3% O_2_, 5% CO_2_) at 37 °C. The cell viability was investigated at 6 h, 12 h, 24 h, and 48 h using CCK-8 assay. According to the results of the CCK-8 assay, superoxide dismutase (SOD) activity was detected using a Total Superoxide Dismutase Assay Kit with WST-8 (Beyotime, Shanghai, China), while the level of the lipid oxidation product malondialdehyde (MDA) was investigated using a Lipid Peroxidation MDA Assay Kit (Beyotime, China).

### 2.8. Statistical Analysis

All data are expressed as mean ± standard deviation (SD). The data from the five or six groups were compared. Statistical analyses were performed using Origin 2017 software. * *p* < 0.01, ** *p* < 0.05, and *** *p* < 0.001 were considered statistically significant.

## 3. Results

### 3.1. Formation of Nano CPO@SiO_2_ with Core–Shell Structure

Currently, the method for preparing CPO particles is well established, but their size is usually at the micron level. Additionally, CPO microspheres do not easily disperse homogeneously in water. To overcome these issues, we prepared CPO nanoparticles using PEG as a dispersant, following Khodaveisi’s report [43]. The selection of PEG as the solvent is a key factor in ensuring the nanoscale size of the prepared CPO particles. First, PEG has a higher viscosity than water, which promotes the dispersion of the prepared CPO nanoparticles and prevents sedimentation during prolonged preparation. Second, PEG prevents the possible dissolution and recrystallization of smaller CPO particles onto larger ones due to the use of water as a solvent (a phenomenon known as the Ostwald ripening effect), which would otherwise lead to an increase in particle size. Based on these advantages, the core–shell structure CPO@SiO_2_ was prepared by coating the SiO_2_ onto the CPO surface. In this system, the CPO core serves as the oxygen source and the SiO_2_ shell layer reduces the contact of CPO with water and further modulates the oxygen generation rate.

The chemical structures of the prepared CPO@SiO_2_ were investigated via FTIR and XRD. The adsorption band of the O-O peak at 867 cm^−1^ in CPO suggested that the CPO had been successfully synthesized [42,44,45]. The stretching vibration of the Si-O-Si appeared at 1068 cm^−1^ in CPO@SiO_2_-1.0T (Figure 1A) [17,46,47,48]. These results suggested the formation of CPO@SiO_2_ nanoparticles. In addition, the asymmetrical stretching vibration and symmetric stretching vibration absorption bands of O-Ca-O appeared at 1423 and 1489 cm^−1^ in both CPO and CPO@SiO_2_ [45,49], which may come from CaO_2_ and the calcium carbonate. The small peak at 1608 cm^−1^ is from the CO_3_^2−^ of calcium carbonate [49,50], produced by the reaction of CPO with carbon dioxide during the preparation and preservation process. Similarly, as shown in Figure 1B, obvious characteristic diffraction peaks of CPO were observed at 30.15°, 35.60°, 47.30°, and 52.94° [43,44,51,52]. Owing to the formation of the SiO_2_ layer, the intensity of these diffraction peaks obviously decreased in the CPO@SiO_2_-1.0T sample. Moreover, the typical dispersion peak of amorphous silica at 22.93° could be observed in CPO@SiO_2_-1.0T [53,54,55]. As shown in Figure 1C, the content of CaO_2_ in CPO was 60–70%. As expected, the CaO_2_ content obviously decreased to 1.8–16.4 wt% in CPO@SiO_2_, owing to the introduction of the SiO_2_ shell layer. However, the content of CaO_2_ in CPO@SiO_2_-0.3T to CPO@SiO_2_-1.0T slightly increased. As shown in Figure S1, a clear spherical distribution of Si and O elements was observed from the mapping image of single nanoparticles in CPO@SiO2-0.5T and CPO@SiO2-1.0T. Ca, however, is too close to Si in atomic number and cannot be observed because it was wrapped in the SiO_2_ shell.

The TEM analysis revealed that bare CPO exhibited an irregular morphology with partial agglomeration, displaying individual particles ranging from 20 to 30 nm in size (Figure S2). In comparison, CPO@SiO_2_-0.1T and CPO@SiO2-0.2T showed significant agglomeration, to the extent that the distinct morphology of individual particles became indiscernible in the TEM images. Notably, with a progressive increase in SiO_2_ shell thickness, the nanoparticles developed well-defined spherical morphologies (Figure 1D). Moreover, the core–shell structure became clearly visible CPO@SiO2-0.3T/0.5T/1.0T/2.0T nanoparticles, as evidenced by the distinct contrast between the inner CPO core and outer SiO_2_ shell in the TEM images. Furthermore, the size of the CPO@SiO_2_ nanoparticles increased with the increases in the thickness of the SiO_2_ layer. The SEM and TEM images showed that the sizes of CPO@SiO_2_-0.3T and CPO@SiO_2_-0.5T are about 60–70 nm. As tetraethyl orthosilicate is added in higher amounts, the sizes of the CPO@SiO_2_-1.0T and CPO@SiO_2_-2.0T nanoparticles increase to 100–120 nm and 220–240 nm, respectively.

The DLS experiments yielded similar results, as shown in Figure 1E,F. The CPO@SiO_2_-0.3T, CPO@SiO_2_-0.5T, CPO@SiO_2_-1.0T, and CPO@SiO_2_-2.0T nanoparticles exhibited a concentration-dependent size progression, exhibiting hydrodynamic diameters of 205.1 ± 2.3 nm, 215.3 ± 3.1 nm, 260.3 ± 4.7 nm, and 302.5 ± 5.6 nm, respectively. These systems maintain monodisperse characteristics, as evidenced by polydispersity indices (PDIs) below 0.15. In contrast, three systems showed divergent behavior: (1) CPO displayed a bimodal distribution (190.1 nm and 5560 nm populations), indicative of hierarchical aggregation, as is consistent with the TEM results; (2) CPO@SiO_2_-0.2T exhibited distinct subpopulations at 122.4 nm and 531.2 nm; and (3) CPO@SiO_2_-0.1T showed a trimodal distribution (141.8 nm, 712.4 nm, and 59 nm), with the largest aggregate size matching the CPO control group. The observed size discrepancies between the DLS and TEM measurements arise from the measurement method: DLS measures the hydrodynamic diameter in solution, which includes solvation layers and is inherently larger than TEM’s dry-state measurements. Based on their superior monodispersity, the CPO@SiO_2_-0.3T, CPO@SiO_2_-0.5T, CPO@SiO_2_-1.0T, and CPO@SiO_2_-2.0T formulations were selected for subsequent oxygen generation and biological studies.

### 3.2. The Oxygen-Generating Behaviors of CPO@SiO_2_

The oxygen-generating behavior of the prepared CPO@SiO_2_ nanoparticles occurs through hydrolysis as follows:

CaO_2_ + 2H_2_O → Ca(OH)_2_ + H_2_O_2_ (6)

2H_2_O_2_ → 2H_2_O + O_2_ (7)

**Figure 2:**
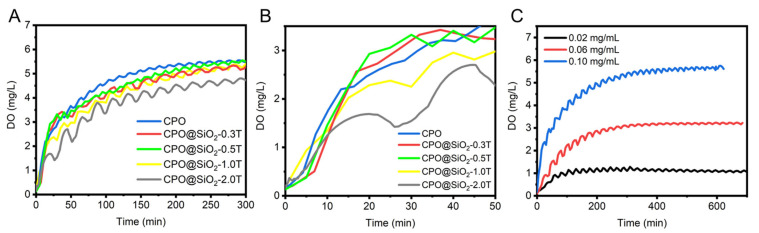
Oxygen-generating behaviors of CPO@SiO_2_. (**A**,**B**) Oxygen-generating curves of CPO and CPO@ SiO_2_ nanoparticles with different shell thicknesses of SiO_2_ shell at 0.10 mg/mL. (**C**) Oxygen-generating curves of CPO@ SiO_2_-1.0T with different concentrations.

In this study, the SiO_2_ shell layer was formed on the surface of CPO to control the production of oxygen. The oxygen-generating profiles of CPO@ SiO_2_ were investigated in a high-pressure airtight container. As shown in Figure 2A, CPO@SiO_2_-0.3T and CPO@SiO2-0.5T exhibited oxygen-generating curves similar to bare CPO. This similarity may stem from two plausible mechanisms: (a) an intact silica encapsulation with low defect density, or (b) subcritical shell thickness insufficient to retard the reaction between CaO_2_ and water. In contrast, the oxygen-generating rate of CPO@SiO_2_-1.0T and CPO@SiO_2_-2.0T obviously decreased during the first 50 min, owing to the SiO_2_ shell layer reducing the reaction rate of water with the CPO core (Figure 2B). While CPO reached its maximum value at 200 min, CPO@SiO_2_-0.3T and CPO@SiO_2_-0.5T delayed this peak at about 250 min. After 300 min, oxygen was released completely in CPO@SiO_2_-1.0T and CPO@SiO_2_-2.0T. Importantly, the SiO_2_ shell layer did not affect the total yield, which remained consistent regardless of the shell thickness. These results suggested that the small amount of SiO_2_ shell layer did not influence the production of oxygen. The total oxygen output could be controlled by adjusting the CPO@SiO_2_ concentration, yielding final dissolved oxygen levels of 1.07 ± 0.5, 3.12 ± 0.3, and 5.34 ± 0.9 mg/L when the concentration of CPO@SiO_2-_1.0T was 0.02 mg/mL, 0.06 mg/mL, and 0.10 mg/mL, respectively (Figure 2C). Therefore, the oxygen-generating behaviors of the CPO@SiO_2_ could be controlled by adjusting the thickness of the SiO_2_ shell layer and the nanoparticle concentration. This phenomenon is consistent with the work of Suvarnapathaki et al., who developed CaO_2_-polycaprolactone (PCL) scaffolds showing concentration-dependent oxygen release [56]. In addition, existing oxygen-releasing systems showed release durations of 6 to 48 h [57,58]. Our CPO@SiO_2_ also achieved extended-duration oxygen generation (>5 h) in aqueous environments when the SiO_2_ thickness and nanoparticle concentration were adjusted (Figure 2A). Notably, Mohammed et al. demonstrated superior sustainability using polylactic acid (PLA)/CPO showing three-day continuous oxygen release [59]. Naturally, the oxygen production duration across studies cannot be precisely compared, owing to variations in measurement techniques and experimental conditions. However, these findings highlight the potential for the future optimization of CPO-based formulations through hydrogel/scaffold integration to meet specific therapeutic oxygen delivery requirements.

### 3.3. Cytocompatibility of CPO@SiO_2_ Nanoparticles

**Figure 3:**
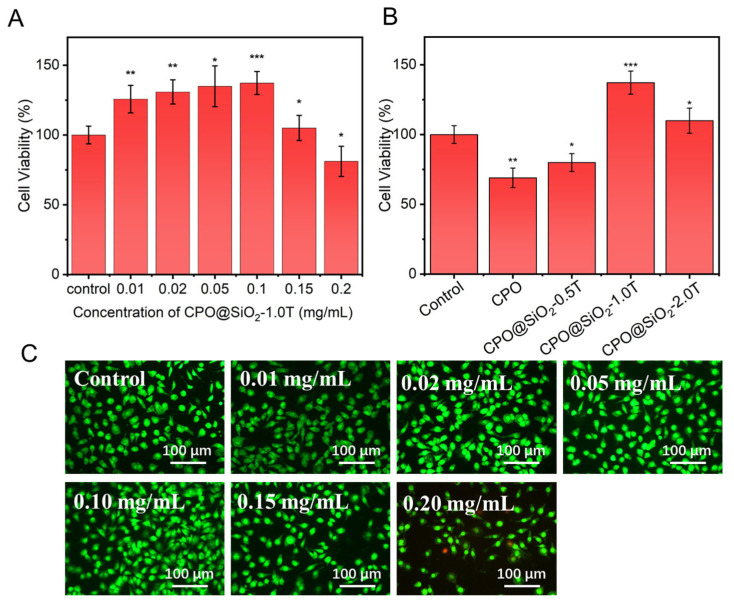
The cell viability of L929 cells cultured in the presence of (**A**) CPO@ SiO_2_-1.0T with different concentrations and (**B**) 0.1 mg/mL of CPO@SiO_2_ nanoparticles with different shell thicknesses (* *p* < 0.05, ** *p* < 0.01, *** *p* < 0.001). (**C**) AO/PI staining images of L929 cells cultured in the presence of CPO@SiO_2_-1.0T with different concentrations.

The cytotoxicity of the prepared CPO@SiO_2_ nanoparticles was evaluated using L929 cells via a CCK-8 assay. As shown in Figure 3A, cell viability gradually increased with the increase in the CPO@SiO_2_-1.0T concentration up to 0.1 mg/mL, then decreased at 0.15 mg/mL. These results indicated that the SiO_2_ shell layer significantly enhanced biocompatibility. The maximum cell viability was achieved at a CPO@SiO_2_-1.0T concentration of 0.1 mg/mL. This phenomenon can be explained as follows: when the nanoparticle concentration was below 0.1 mg/mL, CPO@SiO_2_ continuously generated oxygen during cell culture. The oxygen production increased with the nanoparticle concentration, providing essential nutrients for cell proliferation. However, excessive nanoparticle concentrations adversely affected cellular growth. At 0.2 mg/mL, the nanoparticles exhibited cytotoxicity, resulting in cell viability lower than the control group. The AO/PI staining results were consistent with these findings. As shown in Figure 3C, the number of live cells gradually increased with the concentration of CPO@SiO_2_-1.0T up to 0.1 mg/mL (Figure 3C), while some dead cells could be observed at 0.15 mg/mL due to the production of excess oxygen.

The thickness of the SiO_2_ shell layer was the main factor influencing cytotoxicity. As shown in Figure 3B, the cell viability in the CPO group was obviously lower than that of the control, which may have been caused by the rapid release of oxygen. Although the cell viability in the CPO@SiO2-0.5T group slightly increased compared to that in the bare CPO group, it still showed certain cytotoxicity because of the small SiO_2_ layer. As expected, the CPO@SiO_2_-1.0T and CPO@SiO_2_-2.0T showed excellent biocompatibility, with cell viability significantly exceeding the control group. This enhancement resulted from the suitable oxygen stimulation of cell proliferation. Under normoxic conditions, the CPO@SiO_2_-1.0T released more oxygen, thereby promoting cell reproduction and enhancing cell activity. In contrast, the CPO@SiO_2_-2.0T group released a relatively low amount of oxygen, which had a weaker effect on cell promotion than the CPO@SiO_2_-1.0T group, so the cell activity was more consistent with the control group.

### 3.4. Effect of CPO@SiO_2_ on Oxidant Stress Damages in Cells

Hypoxia is typically the main microenvironment at the lesion site following tissue damage and within large-scale engineered tissue, a state that could induce oxidative stress damage to both host and implanted cells. In this study, L929 cells were cultured in a hypoxic incubator at 3% O_2_ to establish an oxidative stress model. The protective effects of CPO@SiO_2_ on cell survival were investigated using the CCK-8 assay.

**Figure 4:**
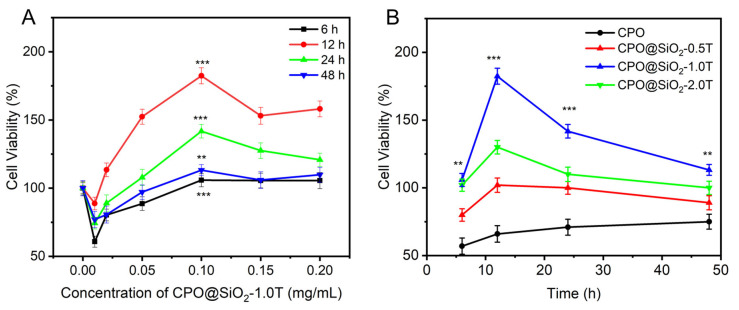
The viability of cells cultured under hypoxia for 48 h in the presence of (**A**) CPO@SiO_2-_1.0T nanoparticles with different concentrations and (**B**) CPO@SiO_2_ nanoparticles with different shell thicknesses at 0.1 mg/mL (** *p* < 0.05, *** *p* < 0.001).

As shown in Figure 4A, the cell viability gradually increased with the increase in CPO@ SiO_2_-1.0T concentrations up to 0.1 mg/mL; then, it gradually decreased while remaining higher than the control group. This occurred because the generated oxygen content increased with the CPO@ SiO_2_-1.0T concentration, which progressively alleviated the hypoxia of cells. It should be noted that the cell viability at 0.01 mg/mL was lower than that of the control, likely due to residual catalase activity causing cell damage at a low concentration of CPO@SiO_2_-1.0T nanoparticles. With the increase in CPO@ SiO_2_-1.0T concentrations, most of the catalase was used to decompose the H_2_O_2_ produced by CPO to generate O_2_ [23]. For all tested concentrations of CPO@SiO_2_-1.0T, cell viability exhibited distinct time-dependent characteristics under hypoxic conditions. The maximum viability was achieved at 12 h, followed by a gradual decline. This temporal pattern can be attributed to the sustained oxygen generation by CPO@SiO_2_-1.0T during the initial 12 h period, which effectively mitigated hypoxia-induced cellular damage. Beyond this time point, the diminishing oxygen generation capacity correlated with reduced cell survival rates, consistent with our oxygen generation profile measurements.

Meanwhile, the cell viability also depended on the thickness of the SiO_2_ shell (Figure 4B). Cells inevitably contacted CPO directly due to the lack of a protective SiO_2_ layer; therefore, the cell viability in the CPO group was lower than that in the control. The CPO@SiO_2_-0.5T group exhibited similar viability to bare CPO. Under hypoxic conditions, the slower oxygen release rate in the CPO@SiO_2_-2.0T group would result in inadequate oxygen supply during the initial hypoxic phase, preventing effective protection against hypoxia-induced damage. Although the CPO@SiO_2_-2.0T group had a longer oxygen release duration, the subsequent oxygen release was insufficient to fully repair the damaged cells. Consequently, the CPO@SiO_2_-2.0T group displayed lower cell activity compared to the CPO@SiO_2_-1.0T group. This highlights that protecting hypoxic cells requires attention not only to the duration of oxygen supply but also to minimizing initial damage [60].

### 3.5. The Effect of CPO@SiO_2_ on ROS Expression Under Hypoxia

**Figure 5:**
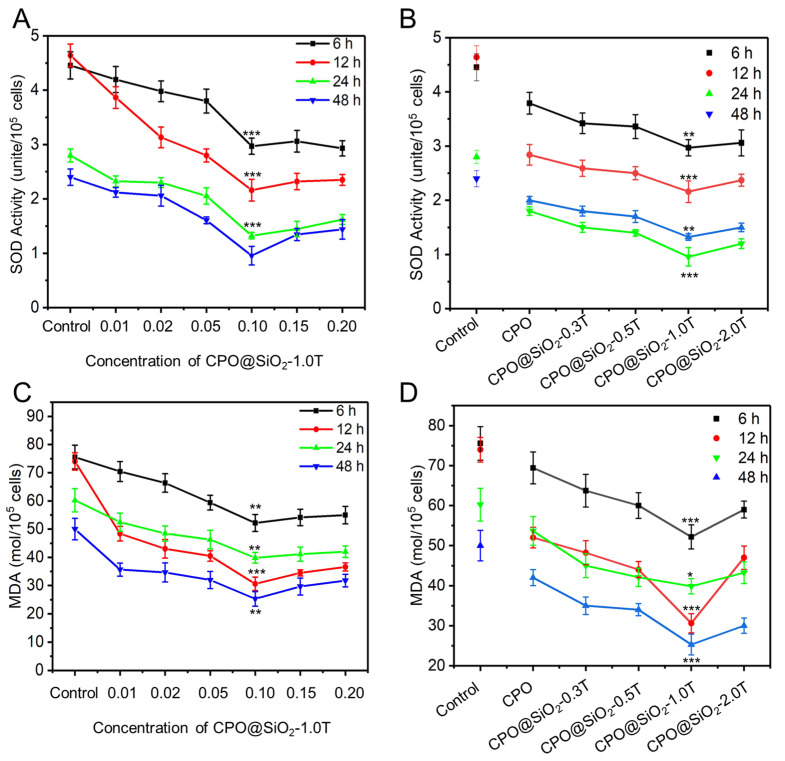
The SOD activity of L929 cells cultured within 48 h in the presence of (**A**) CPO@SiO_2_ nanoparticles with different concentrations and (**B**) CPO@SiO_2_-1.0T nanoparticles with different shell thicknesses at 0.1 mg/mL. The MDA level of L929 cells cultured within 48 h in the presence of (**C**) CPO@SiO_2_ nanoparticles with different concentrations and (**D**) CPO@SiO_2_-1.0T nanoparticles with different shell thicknesses at 0.1 mg/mL. (* *p* < 0.01, ** *p* < 0.05, *** *p* < 0.001).

It is generally believed that oxidative stress under hypoxia produces a large number of ROS, which impairs the normal physiological functions of cells. SOD is an essential antioxidant enzyme that protects cells from ROS [61]. The hypoxic microenvironment led to increased SOD expression levels. The addition of CPO@SiO_2_-1.0T can obviously reduce SOD activity. As shown in Figure 5A, the SOD activity of the cells in the control group was higher than that of all the CPO@SiO_2_-1.0T groups at different concentrations across the four time points. With the increase in CPO@SiO_2_-1.0T concentration, the SOD activity showed a decreasing trend, reaching the lowest value at 0.1 mg/mL. Comparing the four curves at different times, we observed that the SOD activity decreased over time. This may have been due to the continuous release of oxygen, which alleviated hypoxia. In addition, SOD activity in the CPO group was also lower than that in the control group, indicating that oxygen production in the CPO core could relieve hypoxia and thus reduce SOD activity. With the increase in shell thickness, SOD activity gradually decreased until CPO@SiO_2_-1.0T, reaching the lowest value, and then it increased slightly in CPO@SiO_2_-2.0T (Figure 5B). This also verified the protective effect of the SiO_2_ shell on cells. As for CPO@SiO_2_-2.0T, its SOD activity was slightly higher than the CPO@SiO2-1.0T group, which may be due to the slow oxygen production rate, failing to alleviate hypoxia in the early stage.

In biological organisms, free radicals induce lipid peroxidation, with MDA being the final oxidation product. Therefore, the expression levels of MDA can reflect the degree of lipid peroxidation in the cells and indirectly reflect the degree of cell damage [62]. As shown in Figure 5C, cells in CPO@SiO_2_-1.0T with a concentration of 0.1 mg/mL showed the lowest MDA levels, indicating reduced lipid peroxidation and minimal cell damage. Furthermore, at the same concentration, the cells in CPO@SiO_2_-1.0T consistently yielded the lowest MDA levels (Figure 5D). Unlike the trend observed in SOD activity, MDA levels did not exhibit a gradual decline over time.

## 4. Conclusions

In this study, a series of core–shell-structured CPO@SiO_2_ nanoparticles were prepared to alleviate the hypoxic microenvironment. The CPO core served as an oxygen-generating source, and the SiO_2_ shell layer functioned to isolate the CPO core from water and regulate oxygen-generating behaviors. The results suggest that the SiO_2_ shell thickness could be modulated by adjusting the amount of TEOS. The CPO@SiO_2_ nanoparticles exhibited sizes ranging from 205 to 302 nm with narrow PDIs. The SiO_2_ shell layer significantly decreased the particle agglomeration and effectively modulated oxygen generation by adjusting the SiO_2_ shell thickness. In addition, the prepared CPO@ SiO_2_ nanoparticles showed good biocompatibility. Furthermore, they were able to alleviate the hypoxic microenvironment, suppress intracellular ROS, reduce oxidative stress damage, and improve cell survival under hypoxic conditions.

## Data Availability

The original contributions presented in the study are included in the article and Supplementary Materials. Further inquiries can be directed to the corresponding authors.

## Supplementary Materials

The following supporting information can be downloaded at: https://www.mdpi.com/article/10.3390/ma18112568/s1, Figure S1. TEM bright field and mapping of CPO@ SiO_2_-0.5T and CPO@SiO_2_-1.0T nanoparticles. Figure S2. TEM images of CPO, CPO@SiO_2_-0.1T, and CPO@SiO_2_-0.2T nanoparticles.

## References

[1] Willemen N.G.A., Hassan S., Gurian M., Li J., Allijn I.E., Leijten J. (2021). Oxygen-Releasing Biomaterials: Current Challenges and Future Applications. Trends Biotechnol..

[2] Hao T., Li J., Yao F., Dong D., Wang Y., Yang B., Wang C. (2017). Injectable Fullerenol/Alginate Hydrogel for Suppression of Oxidative Stress Damage in Brown Adipose-Derived Stem Cells and Cardiac Repair. ACS Nano.

[3] Bialik S., Geenen D., Sasson I., Cheng R., Horner J.W., Evans S.M., Lord E., Koch C.J., Kitsis R.N. (1997). Myocyte Apoptosis During Acute Myocardial Infarction in The Mouse Localizes to Hypoxic Regions but Occurs Independently of p53. J. Clin. Investig..

[4] Juul S. (2002). Erythropoietin in the Central Nervous System, and Its Use to Prevent Hypoxic-Ischemic Brain Damage. Acta Paediatr..

[5] Ashok B.S., Ajith A., Sivanesan S. (2017). Hypoxia-Inducible Factors as Neuroprotective Agent in Alzheimer’s Disease. Clin. Exp. Pharmacol. Physiol..

[6] Chung H.M., Won H., Sung J. (2009). Responses of Adipose-Derived Stem Cells During Hypoxia: Enhanced Skin-Regenerative Potential. Expert Opin. Biol. Ther..

[7] Nys K., Maes H., Dudek A.M., Agostinis P. (2011). Uncovering the Role of Hypoxia Inducible Factor-1α In Skin Carcinogenesis. Biochim. Biophys. Acta.

[8] Zhang Z., Rong Z., Wu G., Wang Y., Tan Z., Zheng J., Jin Y., Liang Z., Liu C., Guo J. (2021). Gelatin-CaO_2_/SAP/PLGA Composite Scaffold Enhances the Reparation of Critical-Sized Cranial Defects by Promoting Seed Cell Survival. Appl. Mater. Today.

[9] Pedraza E., Coronel M., Fraker C.K., Ricordi C., Stabler C. (2012). Preventing Hypoxia-Induced Cell Death in Beta Cells and Islets via Hydrolytically Activated, Oxygen-Generating Biomaterials. Proc. Natl. Acad. Sci. USA.

[10] Farris A., Rindone A., Grayson W. (2016). Oxygen Delivering Biomaterials for Tissue Engineering. J. Mater. Chem. B.

[11] Li Z., Guo X., Guan J. (2012). An Oxygen Release System to Augment Cardiac Progenitor Cell Survival and Differentiation Under Hypoxic Condition. Biomaterials.

[12] Abdi S., Choi J., Lau H., Lim J. (2013). Controlled Release of Oxygen From PLGA-Alginate Layered Matrix and Its In Vitro Characterization on the Viability of Muscle Cells Under Hypoxic Environment. Tissue Eng. Regen. Med..

[13] Niu H., Li C., Guan Y., Dang Y., Li X., Fan Z., Shen J., Ma L., Guan J. (2020). High Oxygen Preservation Hydrogels to Augment Cell Survival Under Hypoxic Condition. Acta Biomater..

[14] Lin L., Huang T., Song J., Ou X., Wang Z., Deng H., Tian R., Liu Y., Wang J., Liu Y. (2019). Synthesis of Copper Peroxide Nanodots for H_2_O_2_ Self-Supplying Chemodynamic Therapy. J. Am. Chem. Soc..

[15] He J., Fu J., Qi C., Lin J., Huang P. (2021). Metal peroxides For Cancer Treatment. Bioact. Mater..

[16] Cassell O., Hofer S., Morrison W., Knight K. (2002). Vascularisation of Tissue-Engineered Grafts: The Regulation of Angiogenesis In Reconstructive Surgery and In Disease States. Br. J. Plast. Surg..

[17] Rastinfard A., Nazarpak M., Moztarzadeh F. (2018). Controlled Chemical Synthesis of CaO_2_ Particles Coated with Polyethylene Glycol: Characterization of Crystallite Size and Oxygen Release Kinetics. RSC Adv..

[18] Yeh C., Wang R., Chang W., Shih Y. (2018). Synthesis and Characterization of Stabilized Oxygen-Releasing CaO_2_ Nanoparticles for Bioremediation. J. Environ. Manag..

[19] Lee E., Jung J., Alam Z., Yi H., Kim H., Choi J., Hurh S., Kim Y., Jeong J., Yang J. (2018). Effect of An Oxygen-Generating Scaffold on the Viability and Insulin Secretion Function of Porcine Neonatal Pancreatic Cell Clusters. Xenotransplantation.

[20] Mizukami Y., Takahashi Y., Shimizu K., Konishi S., Takakura Y., Nishikawa M. (2021). Calcium Peroxide-Containing Polydimethylsiloxane-Based Microwells for Inhibiting Cell Death in Spheroids through Improved Oxygen Supply. Biol. Pharm. Bull..

[21] McQuilling J., Sittadjody S., Pendergraft S., Farney A., Opara E. (2017). Applications of Particulate Oxygen-Generating Substances (POGS) in the Bioartificial Pancreas. Biomater. Sci..

[22] Lu Z., Jiang X., Chen M., Feng L., Kang Y.Z. (2019). An oxygen-Releasing Device to Improve the Survival of Mesenchymal Stem Cells in Tissue Engineering. Biofabrication.

[23] Kang J.I., Park K.M., Park K.D. (2019). Oxygen-Generating alginate Hydrogels as a Bioactive Acellular Matrix for Facilitating Wound Healing. J. Ind. Eng. Chem..

[24] Motealleh M., Kehr N.S. (2020). Injectable Oxygen-Generating Nanocomposite Hydrogels with Prolonged Oxygen Delivery for Enhanced Cell Proliferation Under Hypoxic and Normoxic Conditions. J. Mater. Chem. B.

[25] Montesdeoca C.Y.C., Afewerki S., Stocco T.D., Corat M.A.F., Paula M.M.M., Marciano F.R., Lobo O. (2020). Oxygen-Generating Smart Hydrogels Supporting Chondrocytes Survival in Oxygen-Free Environments. Colloids Surf. B Biointerfaces.

[26] Alemdar N., Leijten J., Camci-Unal G., Hjortnaes J., Ribas J., Paul A., Mostafalu P., Gaharwar A.K., Qiu Y., Sonkusale S. (2017). Oxygen-Generating Photo-Cross-Linkable Hydrogels Support Cardiac Progenitor Cell Survival by Reducing Hypoxia-Induced Necrosis. ACS Biomater. Sci. Eng..

[27] Park S., Park K.M. (2018). Hyperbaric oxygen-generating hydrogels. Biomaterials.

[28] Tamaddon M., Gilja H., Wang L., Romandini I., Filippis I.R.D., Zaffagnini S., Filardo J. (2020). Osteochondral Scaffolds for Early Treatment of Cartilage Defects in Osteoarthritic Joints: From Bench to Clinic. Biomater. Transl..

[29] Xu X., Song J. (2020). Segmental Long Bone Regeneration Guided by Degradable Synthetic Polymeric Scaffolds. Biomater. Transl..

[30] Ai C., Lee Y.H.D., Tan X.H., Tan S.H.S., Hui J.H.P., Goh J.C.-H. (2021). Osteochondral tissue engineering: Perspectives for Clinical Application and Preclinical Development. J. Orthop. Transl..

[31] Li L., Yu F., Zheng L., Wang R., Yan W., Wang Z., Xu J., Wu J., Shi D., Zhu L. (2019). Natural Hydrogels for Cartilage Regeneration: Modification, Preparation and Application. J. Orthop. Transl..

[32] Hsieh T.E., Lin S.J., Chen L.C. (2020). Optimizing an Injectable Composite Oxygen-Generating System for Relieving Tissue Hypoxia. Front. Bioeng. Biotechnol..

[33] Abudula T., Gauthaman K., Hammad A.H., Chen C.C., Lai P.L., Huang C.C. (2020). Oxygen-Releasing Antibacterial Nanofibrous Scaffolds for Tissue Engineering Applications. Polymers.

[34] Oh S.H., Ward C.L., Atala A., Yoo J.J., Harrison B.S. (2009). Oxygen Generating Scaffolds for Enhancing Engineered Tissue Survival. Biomaterials.

[35] Lv X.G., Li Z., Chen S.Y., Xie M.K., Huang J.W., Peng X.F., Yang R.X., Wang H.P., Xu Y.M., Feng C. (2016). Structural and Functional Evaluation of Oxygenating Keratin/Silk Fibroin Scaffold and Initial Assessment of Their Potential for Urethral Tissue Engineering. Biomaterials.

[36] Erdem A., Darabi M.A., Nasiri R., Sangabathuni S., Ertas Y.N., Alem H., Hosseini V., Shamloo A., Nasr A.S., Ahadian S. (2020). 3D Bioprinting: 3D Bioprinting of Oxygenated Cell-Laden Gelatin Methacryloyl Constructs. Adv. Healthcare Mater..

[37] Ortega J.A., Sirois C.L., Memi F., Glidden N., Zecevic N. (2017). Oxygen Levels Regulate the Development of Human Cortical Radial Glia Cells. Cerebral. Cortex..

[38] Wang R., Sun Y.F., Wang H., Liu T., Shavandi A., Nie L., Yunusov K.E., Jiang G.H. (2024). Core-Shell Structured Microneedles with Programmed Drug Release Functions for Prolonged Hyperuricemia Management. J. Mater. Chem. B.

[39] Wu X., Han X., Guo Y., Liu Q., Sun R., Wen Z., Dai C. (2023). Application Prospect of Calcium Peroxide Nanoparticles in Biomedical Field. Rev. Adv. Mater. Sci..

[40] Hwang U., Kim J., Kim N.K., Choi K., Chung J.Y., Kim T., Suhr J., Nam J.D. (2022). Surface Charge Control of Hierarchical Ceria/Silica Hybrid Shells for Enhanced Dispersion Stability. Appl. Surf. Sci..

[41] Kim H.S., Lee J.H., Mandakhbayar N., Jin G.Z., Kim S.J., Yoon J.Y., Jo S.B., Park J.H., Singh R.K., Jang J.H. (2021). Therapeutic Tissue Regenerative Nanohybrids Self-Assembled from Bioactive Inorganic Core / chitosan Shell Nanounits. Biomaterials.

[42] Wu D., Zhu Z.-Q., Tang H.-X., Shi Z.-E., Kang J., Liu Q., Qi J. (2020). Efficacy-shaping Nanomedicine by loading Calcium Peroxide into Tumor Microenvironment-Responsive Nanoparticles for the Antitumor Therapy of Prostate Cancer. Theranostics.

[43] Khodaveisi J., Banejad H., Afkhami A., Olyaie E., Lashgari S., Dashti R. (2011). Synthesis of Calcium Peroxide Nanoparticles as an Innovative Reagent for in Situ Chemical Oxidation. J. Hazard. Mater..

[44] Madan S.S., Wasewar K.L., Kumar C.R. (2016). Adsorption Kinetics, Thermodynamics, and Equilibrium of α-toluic Acid onto Calcium Peroxide Nanoparticles. Adv. Powder Technol..

[45] Balci B., Aksoy N., Erkurt F.E., Budak F., Basibuyuk M., Zaimoglu Z., Turan E.S., Yilmaz S. (2021). Removal of a Reactive Dye from Simulated Textile Wastewater by Environmentally Friendly Oxidant Calcium Peroxide. Int. J. Chem. React. Eng..

[46] Mendes G., Faria M., Carvalho A., Gonçalves M.C., de Pinho M.N. (2018). Structure of Water in Hybrid Cellulose Acetate-silica Ultrafiltration Membranes and Permeation Properties. Carbohydr. Polym..

[47] Tai X., Ma J., Du Z., Wang W., Wu J. (2013). A Simple Method for Synthesis of Thermal Responsive Silica Nanoparticle/PNIPAAm Hybrids. Powder Technol..

[48] Du Mont J.W., Marquardt A.E., Cano A.M., George S.M. (2017). Thermal Atomic Layer Etching of SiO_2_ by a “Conversion-Etch” Mechanism Using Sequential Reactions of Trimethylaluminum and Hydrogen Fluoride. ACS Appl. Mater. Interfaces.

[49] Ali M., Farooq U., Lyu S., Sun Y., Li M., Ahmad A., Shan A., Abbas Z. (2020). Synthesis of Controlled Release Calcium Peroxide Nanoparticles (CR-nCPs): Characterizations, H_2_O_2_ Liberate Performances and Pollutant Degradation Efficiency. Sep. Purif. Technol..

[50] Xue P., Hou M., Sun L., Li Q., Zhang L., Xu Z., Kang Y. (2018). Calcium-carbonate Packaging Magnetic Polydopamine Nanoparticles Loaded with Indocyanine Green for Near-infrared Induced Photothermal/photodynamic Therapy. Acta Biomater..

[51] Takada S., Hata N., Seino Y., Fujii N., Kikkawa T. (2005). Skeletal Silica Characterization in Porous-silica Low-dielectric-constant Films by Infrared Spectroscopic Ellipsometry. J. Appl. Phys..

[52] Wu B., Su L., Dai X., Chai X. (2017). Development of Montmorillonite-supported Nano CaO_2_ for Enhanced Dewatering of Waste-activated Sludge by Synergistic Effects of Filtration Aid and Peroxidation. Chem. Eng. J..

[53] Wang J., Lu C., Xiong J. (2014). Self-Cleaning and Depollution of Fiber Reinforced Cement Materials Modified by Neutral TiO_2_/SiO_2_ Hydrosol Photoactive Coatings. Appl. Surf. Sci..

[54] Musić S., Filipović-Vinceković N., Sekovanić L. (2011). Precipitation of Amorphous SiO_2_ Particles and Their Properties. Braz. J. Chem. Eng..

[55] Dhanalekshmi K.I., Meena K.S. (2014). Comparison of Antibacterial Activities of Ag@TiO_2_ and Ag@SiO_2_ Core-Shell Nanoparticles. Spectrochim. Acta A Mol. Biomol. Spectrosc..

[56] Suvarnapathaki S., Nguyen M.A., Goulopoulos A.A., Lantigua D., Camci-Unal G. (2021). Engineering Calcium Peroxide Based Oxygen Generating Scaffolds for Tissue Survival. Biomater. Sci..

[57] Seekell R.P., Lock A.T., Peng Y., Cole A.R., Perry D.A., Kheir J.N., Polizzotti B.D. (2016). Oxygen Delivery Using Engineered Microparticles. Proc. Natl. Acad. Sci. USA.

[58] Cook C.A., Hahn K.C., Morrissette-McAlmon J.B., Grayson W.L. (2015). Oxygen Delivery from Hyperbarically Loaded Microtanks Extends Cell Viability in Anoxic Environments. Biomaterials.

[59] Mohammed A., Saeed A., Elshaer A., Melaibari A.A., Memi’c A., Hassanin H., Essa K. (2023). Fabrication and Characterization of Oxygen-Generating Polylactic Acid/Calcium Peroxide Composite Filaments for Bone Scaffolds. Pharmaceuticals.

[60] Pastwińska J., Walczak-Drzewiecka A., Kozłowska E., Harunari E., Ratajewski M., Dastych J. (2022). Hypoxia Modulates Human Mast Cell Adhesion to Hyaluronic *Acid*. Immunol. Res..

[61] Azadmanesh J., Borgstahl G.E.O. (2018). A Review of the Catalytic Mechanism of Human Manganese Superoxide Dismutase. Antioxidants.

[62] Rodríguez-García A., García-Vicente R., Morales M.L., Ortiz-Ruiz A., Martínez-López J., Linares M. (2020). Protein Carbonylation and Lipid Peroxidation in Hematological Malignancies. Antioxidants.

