# Supplementary material for: Preparation and Properties of Calcium Peroxide/Poly(ethylene glycol)@Silica Nanoparticles with Controlled Oxygen-Generating Behaviors
Source: Materials (Basel). 2025 May 30;18(11):2568. doi: 10.3390/ma18112568 (PMC12156953; doi:10.3390/ma18112568)
Supplement: Supplementary file 1 [file materials-18-02568-s001.zip › materials-3560883-supplementary.pdf]

# Supporting Materials

## Preparation and Property of Calcium Peroxide/Poly(ethylene glycol)@Silica Nanoparticles with Controlled Oxygen-Generating Behaviors

Xiaoling Xie [1#,] Xin Sun [2#], Wanming Lin [1]*, Xiaofeng Yang [1], Ruicong Wang [1]

[1] Shanxi University Electronic Science and Technology, Linfen 041000 Peoples R China; xiexl2003@126.com

[2] Zhejiang Institute of Tianjin University, Ningbo 315201, Peoples R China; sunxin_tju@163.com

* Correspondence: linwm1970@126.com;

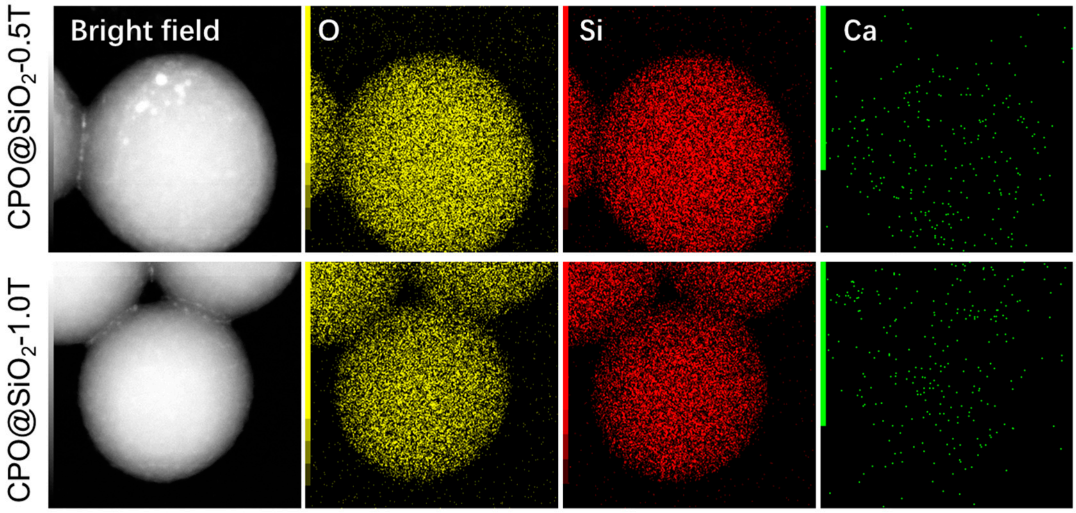


Figure S1TEM bright field and mapping of CPO@ $SiO_2$-0.5T and CPO@$SiO_2$-1.0T nanoparticles.

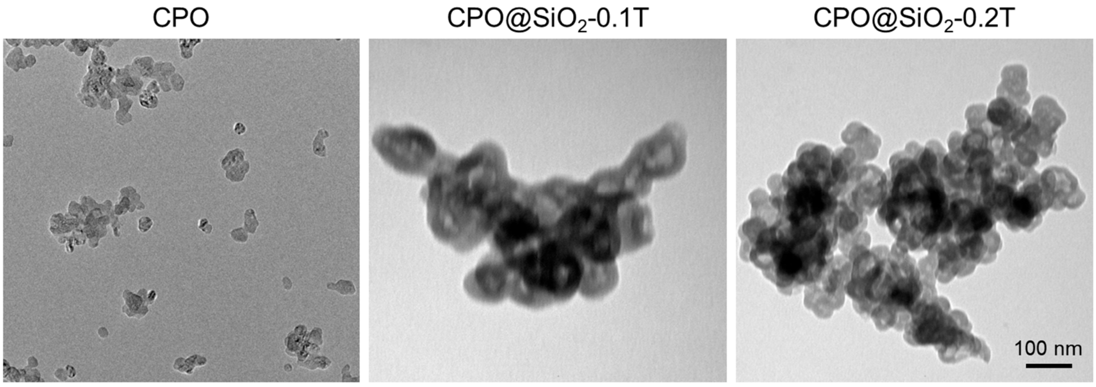


Figure S2 TEM images of CPO, CPO@$SiO_2$-0.1T and CPO@$SiO_2$-0.2T nanoparticles
